# Microglia/Macrophages and CD4^+^CD25^+^ T Cells Enhance the Ability of Injury-Activated Lymphocytes to Reduce Traumatic Optic Neuropathy *In Vitro*

**DOI:** 10.3389/fimmu.2021.687898

**Published:** 2021-08-13

**Authors:** Yiqun Geng, Zhihao Lu, Jitian Guan, Nico van Rooijen, Ye Zhi

**Affiliations:** ^1^Laboratory of Molecular Pathology, Guangdong Provincial Key Laboratory of Infectious Diseases and Molecular Immunopathology, Shantou University Medical College, Shantou, China; ^2^Joint Shantou International Eye Center of Shantou University and the Chinese University of Hong Kong, Shantou University Medical College, Shantou, China; ^3^Department of MRI, the Second Affiliated Hospital of Shantou University Medical College, Shantou, China; ^4^Department of Molecular Cell Biology, Vrije Universiteit Medical Center, Amsterdam, Netherlands; ^5^Department of Anatomy, Shantou University Medical College, Shantou, China

**Keywords:** retinal ganglion cell, microglia/macrophage, inflammation, trauma, CD4+ Cd25+ T cells

## Abstract

Inflammation after acute CNS injury plays a dual role. The interplay between immune cells and inflammatory mediators is critical to the outcome of injured neurons. Microglia/macrophages are the first sensors and regulators of the immune response. We previously found that the enhancement of macrophages on neuron survival does not persist in thymectomized rats. How T lymphocytes and macrophages interact and benefit neuron survival is not fully elucidated. To this point, we introduce and characterize a cell-retina co-culture model that mimics the recruitment of peripheral lymphocytes at the injury site. Three-day post-optic nerve transection (ONT) in Fischer 344 rats, transected retinas were co-cultured with either peripheral lymph node-derived lymphocytes (injury-activated) or from intact rats as the control. The injury-activated lymphocytes preserved retinal ganglion cells (RGCs) and caused extensive retina microglial/macrophage infiltration. CD4^+^CD25^+^ T cells were upregulated in the injury-activated lymphocytes and increased RGC survival, suggesting that CD4^+^CD25^+^ T cells suppressed the cytotoxicity of control lymphocytes. When microglia/macrophages were depleted by clodronate, neuron loss was more extensive, the cytotoxicity of control lymphocytes on RGCs was alleviated, and the neuroprotective effect of injury-activated lymphocytes remain unchanged Cytokine detection showed an increase in IL-6 and TNF-α levels that were reduced with microglia/macrophage depletion. Our results suggest that microglial/macrophage infiltration into axotomized retinas promotes RGC survival by secreting cytokines to induce CD4^+^CD25^+^ T cells and suppress T cell-mediated RGC toxicity. These findings reveal a specific role for microglia/macrophage and CD4^+^CD25^+^ T cells in inflammation after CNS injury, thereby adding to the mechanistic basis for the development of microglial/macrophage modulation therapy for traumatic CNS injury.

## Introduction

Injury to the mature central nervous system (CNS) eventually leads to neuron loss. Concerning injury of the optic nerve, traumatic optic neuropathy or glaucoma results in loss of retinal ganglion cells and loss of vision. This effect is not only caused by immediate disruption, but also by the secondary injury resulting from cascades of inflammatory, metabolic, cellular, and molecular events over time (1). It has been viewed that inflammation after an injury is neurotoxic. However, suppression of neuroinflammatory responses shortly after injury by immune-dampening drugs (e.g., methylprednisolone and progesterone) does not achieve a clinical benefit in human traumatic brain injury (2, 3).

Increasing evidence shows the benefits of the inflammation phase (4). At the early stage of injury, microglia, monocytes, macrophages, neutrophils, and T cells can collectively orchestrate a response that preserves neural tissue (5). Upon CNS injury, both resident and peripheral immune cells are involved, T cells home to the site of injury, and microglia/macrophages are activated. We previously reported that zymosan-induced microglia/macrophage activation improves axotomized retinal ganglion cell (RGC) survival *in vivo* in both F344 rats and experimental autoimmune encephalomyelitis-susceptible Lewis rats, but not in thymectomized rats (6). This indicates that microglia/macrophages participate in crosstalk with T cells to affect the survival of neurons (7), but the process has yet to be elucidated. To this end, we use an axotomized retina and lymph node-derived lymphocyte co-culture model to mimic the peripheral immune cell infiltration, and the consequent interaction with resident microglia/macrophages, to understand how the outcome of axotomized RGCs is affected.

## Materials And Methods

### Animals

F344 male and female rats (6–8 weeks of age) were obtained from Vital River (Beijing, China); the animals were obtained originally from the Charles River Laboratory (Wilmington, MA, USA). Rats were housed at 22°C on a 12 h light/dark cycle and had *ad libitum* access to food and water. All experiments were reviewed and approved by the Shantou University Medical College Animal Experimentation Ethics Committee and carried out following the National Institutes of Health guidelines for the care and use of animals.

### Surgery

The optic nerve transection (ONT) procedure was performed as described previously (8, 9). Briefly, after anesthesia with ketamine and xylazine, the left optic nerve of the rats was exposed through a posterior temporal intraorbital approach and completely transected about 1.5 mm behind the optic disc. The contralateral optic nerve was left intact.

### Lymph Node-Derived Lymphocyte (LNDL) Culture

Rats were euthanized with an overdose of ketamine and xylazine on the third day after ONT. Lymph nodes (axillary, inguinal, and superficial cervical) were harvested, digested, and passed through a cell strainer (100 µm). After counting, 1 × 10^6^ lymphocytes (derived from the three lymph nodes) were cultured for 1 day in Dulbecco’s modified Eagle’s medium (DMEM; Corning, Corning, NY, USA) supplemented with 1% penicillin/streptomycin solution and 10% fetal bovine serum (heat-inactivated) in an incubator with 5% CO_2_ at 37°C to remove most of the attached microglia/macrophages and contaminating fibroblasts. LNDLs harvested from ONT rats were defined as (injury) activated lymphocytes and those from intact rats were defined as control lymphocytes.

### Retinal Explant Co-Culture

Retinas were dissected in cold Hank’s balanced salt solution (HBSS) for retinal explant co-culture according to procedures described previously (6). Briefly, retinas were mounted, onto a nitrocellulose filter paper, with the retinal ganglion cell (RGC) layer uppermost. Activated or control lymphocytes (1 × 10^6^) were then co-cultured in DMEM (n = 5–6 for each group) with retinas for 7 days, with a change of freshly harvested counterpart lymphocytes on the 3^rd^ day.

### Isolation of CD4^+^, CD8^+^, and CD4^+^CD25^+^ Lymphocytes

Lymphocyte subsets were enriched on T cell columns (R&D Systems, Minneapolis, MN, USA) by negative selection. CD8^+^ lymphocytes were removed from the enriched lymphocyte population by incubation with anti-CD8 microbeads, and the negatively selected CD4^+^ lymphocytes were incubated with phycoerythrin (PE)-conjugated anti-CD25 (30 μg/10^8^ cells) in PBS containing 2% fetal bovine serum (heat-inactivated) for 1 hour at room temperature. The cells were then washed and incubated with anti-PE microbeads and subjected to magnetic separation with an AutoMACS. All reagents were from Miltenyi Biotech (Bergisch Gladbach, Germany). The eluted cells from the microbeads were positively selected for CD4^+^CD25^+^ by “panning” to remove most of the microglia/macrophages and contaminating fibroblasts (10). Of the resulting cells, 95% were positive for CD4^+^CD25^+^, as judged by fluorescence-activated cell sorting analysis. For further co-culture, 1 × 10^6^ of each subgroup of cells was selected.

### Microglia/Macrophage Depletion

The use of clodronate-containing liposomes is a widely accepted approach to deplete monocytes/macrophages and microglia. Liposome-encapsulated clodronate liposomes were prepared as previously described (11). Intravenous applications of clodronate liposomes (0.5 ml per 100 g body weight) were administered *via* tail vein right before the ONT procedure (12) and 200 μg/mL of liposomal clodronate was used in the co-culture system.

### Immunohistochemistry

Retinas were fixed with 4% paraformaldehyde for 1 h after removal from culture and transferred to 30% sucrose for at least 4 h. To determine the viability of RGCs and microglia/macrophages, retinas were divided from the nasal to the temporal side. One half was immunostained with primary antibody against mouse anti-βIII-tubulin (TUJ-1, 1:400, Babco, Richmond, CA, USA), and the other half with mouse anti-ED1 (1:200, Serotec, Oxford) followed by incubation with a Cy3-conjugated secondary anti-mouse antibody (1:400, Jackson ImmunoResearch, West Grove, PA, USA) and FITC-conjugated secondary anti-mouse antibody (1:400 Sigma) as secondary antibodies (6, 9, 13). In brief, retinas on the filters were flat-mounted on coverslips and the numbers of surviving RGCs were counted in sample fields with an area of 0.235 × 0.235 mm; 60-80 fields were counted per retina under a fluorescence microscope. The average number per field of βIII-tubulin^+^ RGCs and ED1 was determined respectively.

### Flow Cytometry Determination

Lymphocytes were double-labeled with the following mouse anti-rat dual reagents: CD3-FITC/CD4-RPE; CD3-FITC/CD8-RPE; CD4-FITC/CD25-RPE; CD4-FITC/CD62L-RPE, or an isotype antibody (all from Bio-Rad, Hercules, CA, USA). Changes in expression were examined among three groups: activated lymphocytes, CD3^+^CD4^+^, and CD4^+^CD25^+^ T cells following co-culture with ONT retinas. A total of 1 × 10^6^ cells was collected for each analysis. Cells were washed, fixed, permeabilized, antibody stained, and analyzed by flow cytometry (FACSCalibur, BD Biosciences, Franklin Lakes, NJ, USA), and the percentages of surface markers were recorded.

### Cytometry Bead Array

To examine the Th1/Th2 balance of cytokine production, we used BD™ Cytometric Bead Array Flex Sets (BD Biosciences Franklin Lakes, NJ, USA) to observe the changes of inflammatory cytokines IL-4, IL-6, IL-10, IFN-γ, and TNF-α at different time points after culture for 12h, 24h, 48h or 72h. For this, 1x10^6^ cells were collected in DMEM (Thermo Fisher Scientific Inc, Carlsbad, CA, USA) and placed in a tube. After adding in 20 ng/ml phorbol 12-myristate 13 acetate plus 1 µg/ml ionomycin and culturing at 37°C for 1 hour, 10 ng/ml Brefeldin-A (Millipore Sigma, Saint Louis, MO, USA) was added and cells were cultured at 37°C for 3-5 hours. Then the cells were washed, fixed, permeabilized, and analyzed by flow cytometry

### Cytokine Modulation

Monoclonal anti-rat TNF-α/TNFSF1A TNFR1/2 antibody and anti-rat IL-6 antibody, recombinant rat IL-6 and recombinant rat TNF-α protein (R&D Systems, Minneapolis, MN, USA) were added to the co-culture medium and cells were co-cultured for 7 days. The number of surviving RGCs was counted after immunostaining.

### Statistical Analyses

The sample size was decided based on previous studies (6, 12), and not predetermined by a statistical method. No randomization method was used. Data distribution was assumed to be normal, but this was not formally tested. All tests were two-sided. Error bars indicate the standard deviation of the mean. *P* < 0.05 was considered to indicate statistical significance. Either an unpaired independent T-test or two-way analysis of variance (ANOVA) was used to evaluate the significance of inter-group differences, followed by Bonferroni adjustment to compare mean values among all inter-group comparisons (9, 14, 15), using Prism software version 8.0 (GraphPad, La Jolla, CA, USA).

## Results

### Injury-Activated Lymphocytes Reduce RGC Cytotoxicity in a Manner Consistent With Elevated Microglia/Macrophages

**Figure 1:**
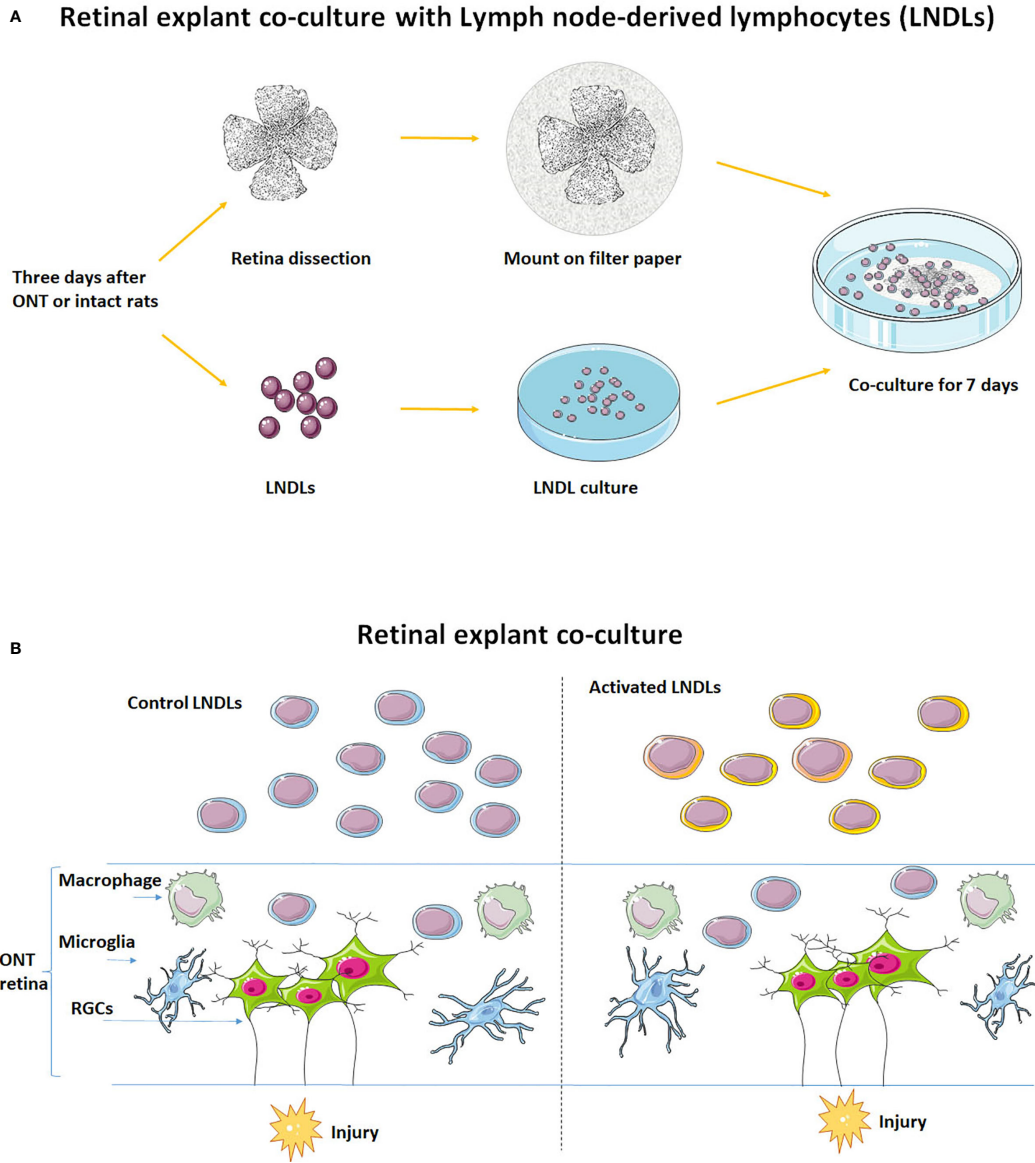
Schematic of the *in vitro* retinal explant and lymphocyte co-culture system. **(A)** Recruitment of LNDLs of F344 rats at the 3^rd^ day after ONT and co-culture with retinal explant for 7 days. **(B)** Illustration of co-culture of rental explant and lymphocytes.

**Figure 2:**
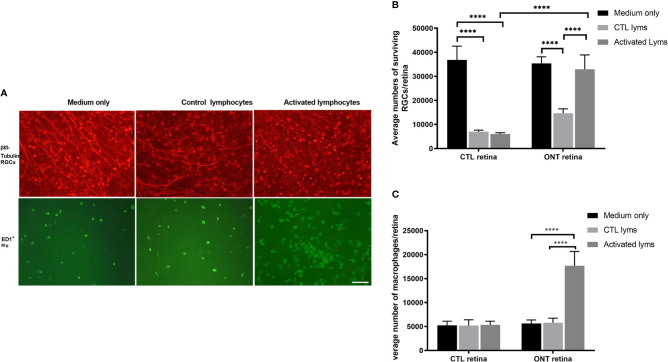
Effect of lymphocyte co-culture on retinal ganglion cell (RGC) survival and macrophage infiltration. **(A)** Representative images of flat-mounted retinas showing β-III tubulin-positive RGCs and ED1-positive microglia/macrophages. Scale bar, 50 µm. **(B)** Quantification of RGCs showed that the co-culture of activated lymphocytes increased the RGCs compared to control lymphocytes co-culture. **(C)** Quantification of microglia/macrophages showed dramatic augmentation in the co-culture of activated lymphocytes compared to other culture conditions. Data are presented as means ± SEM (n=4-6 of each group). ****P < 0.0001. The number of retinas in each group was 4-6.

We co-cultured ONT and control retinas with activated and control lymphocytes for 7 days (**Figures 1A, B**). For ONT retinas, compared with the culture medium, co-culture with control lymphocytes reduced RGC survival (35,381 ± 2,744 *vs* 14,699 ± 1,790, respectively; *P* < 0.0001). In contrast, co-culture with activated lymphocytes showed sustained RGCs (32,927 ± 5,960), with survival being very similar to that in the medium-only culture (*P* = 0.6882). Therefore, co-culture with activated lymphocytes elicited significant improvement in viability compared with co-culture with control lymphocytes (**Figures 2A, B**).

The microglia/macrophages were evaluated, after co-culture, by ED1 immunostaining in the other half of the retina. ED1 expression was elevated dramatically in the activated lymphocyte co-culture group (5,785 ± 964 *vs*. 1, 7671 ± 2,992, *P* < 0.0001), whereas the number of microglia/macrophages in either the medium only group or control lymphocyte co-culture group remained unchanged (**Figure 2C**).

### Augmentation of CD4^+^CD25^+^ T cells in Activated Lymphocyte Cultures Improves RGC Survival

**Figure 3:**
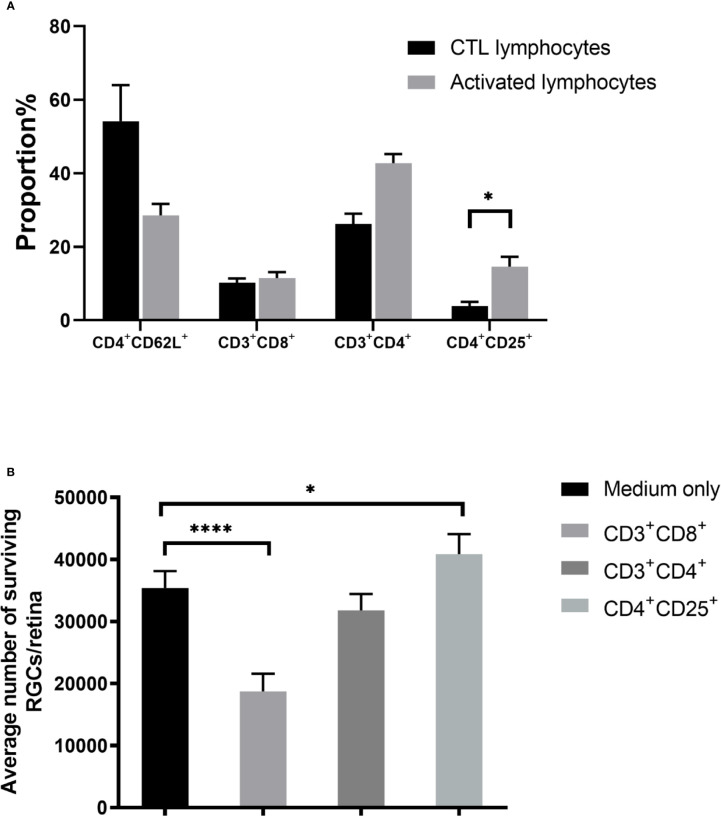
Effect of T cell subsets on retinal ganglion cell (RGC) survival. **(A)** Compared with the control T cell co-culture, co-culture with activated T cells showed upregulation of CD4^+^CD25^+^ T cells. **(B)** Co-culture of retinal explants with purified subgroups of activated T cells showed that CD3^+^CD8^+^ T cell co-culture decreased RGC survival, whereas RGC survival with CD3^+^CD4^+^ T cell co-culture was similar to that of control. RGC survival was even higher when co-cultured with CD4^+^CD25^+^ T cells than in the medium-only control (n=4-5 for each group). *P < 0.05, ****P < 0.0001.

Our initial findings indicated that activated lymphocytes were responsible for RGC survival (6). Therefore, we analyzed the proportion of lymphocyte subsets after co-culture, including CD4^+^CD62L^+^ (central memory T), CD3^+^CD4^+^ (effector T), CD3^+^CD8^+^ (cytotoxic T), and CD4^+^CD25^+^ T cells. Both CD3^+^CD4^+^ effector (~1.6-fold) and CD4^+^CD25^+^ T cells (~3.8-fold) cells were significantly upregulated. Notably, the percentage of CD4^+^CD25^+^ T cells in the control lymphocyte co-culture, which was 3.84 ± 1.16%, rose to 14.63 ± 2.63% in the activated lymphocyte co-culture (*P* = 0.019). However, the proportion of CD3^+^CD8^+^ cells, which are assumed to be cytotoxic (16, 17), was not significantly different between control and activated lymphocytes after being co-cultured with ONT retinal explants (**Figure 3A**).

To determine the effect of CD4^+^CD25^+^ T cells numbers on RGC survival, we segregated the activated lymphocyte mixture into subsets of CD3^+^CD4^+^, CD3^+^CD8^+^, and CD4^+^CD25^+^ T cells, then co-cultured ONT retinas with each subset, followed by enumeration of viable RGCs. Compared with the medium only culture (35,381 ± 2,744), RGCs in the CD3^+^CD8^+^ cell co-culture decreased (18,732 ± 2,853, *P* < 0.0001) but increased in the CD4^+^CD25^+^ T cells co-culture (40,837 ± 3,233, *P* = 0.0287), whereas no significant difference was found between the CD3^+^CD4^+^ cell co-culture and the medium only (**Figure 3B**).

### Depletion of Microglia/Macrophages Affects RGC Survival

**Figure 4:**
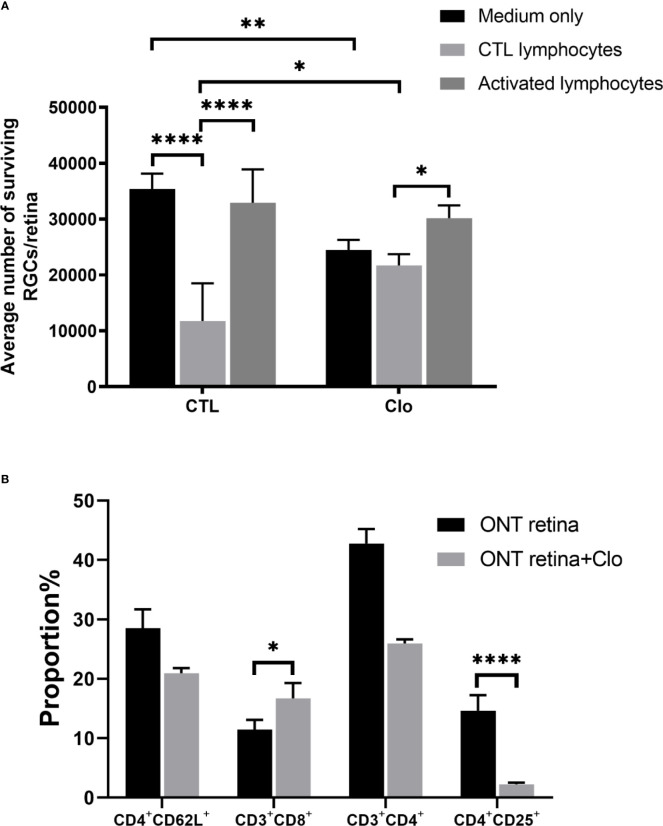
Microglia/macrophages on the viability of RGCs **(A)** With the depletion of microglia/macrophages by clodronate, the number of RGCs was reduced in the medium-only group, slightly elevated in the CTL lymphocyte co-culture group, and unchanged in the activated lymphocyte co-culture group. **(B)** T cell subset analysis showed that CD3^+^CD8^+^ cell numbers increased and CD4^+^CD25^+^ cell numbers decreased. Clo=clodronate (n=4-6 for each group). *P < 0.05, **P < 0.01, ****P < 0.0001.

Our findings also indicated that the status of microglia/macrophages affects RGC viability. We used clodronate liposomes to deplete the resident monocytes and microglia/macrophages in the retinas. Compared with RGCs on control retinas (14,699 ± 1,790) clodronate-treated retinas showed extenuated cytotoxicity when co-cultured with control lymphocytes (21,700 ± 2,316) (*P* = 0.0122), but the difference was not statistically significant compared with that in the activated lymphocyte co-culture (32,927 ± 6,410 *VS* 30,166 ± 2,286, *P* = 0.31) (**Figure 4A**). Subtype analysis of the co-cultured activated lymphocytes showed that when microglia/macrophages were depleted, the proportions CD4^+^CD25^+^ T cells were dramatically lower (2.13 ± 0.208%) than that in the non-depleted counterpart 14.87 ± 3.16% (*P*<0.0001) (**Figure 4B**).

### IL-6 and TNF-α Contribute to RGC Survival

**Figure 5:**
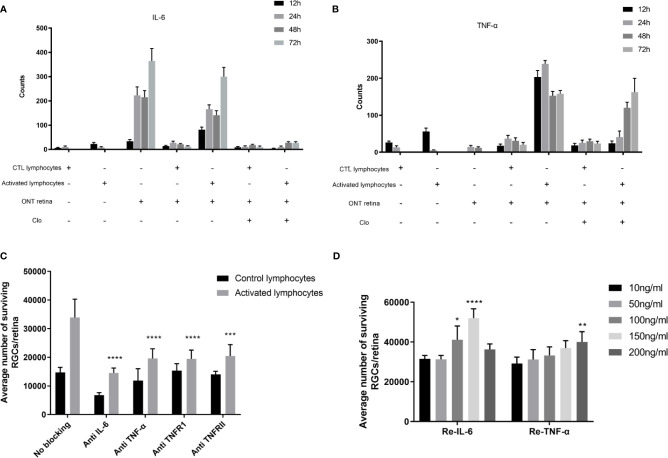
Detection and effect of IL-6 and TNF-α **(A, B)** IL-6 and TNF-α detection by cytometric bead array in the culture medium following culture under different conditions for varying times. IL-6 was elevated in axotomized retinas only and with activated lymphocyte co-culture. Microglia/macrophage depletion reduced the secretion. TNF-α was secreted in the medium of retinas and was slightly suppressed in co-culture with activated lymphocytes in the first 24 h but returned to peak afterward following microglia/macrophage depletion. **(C)** Compared to non-blocked activated lymphocyte co-culture, antibodies against IL-6, TNF-α, TNFR1, and TNFR2 reduced RGC viability. **(D).** Recombinant IL-6 and TNF- α increased the RGC viability in a dose-dependent manner (n=4-5 for each group). *P < 0.05, **P < 0.01, ***P < 0.001, ****P < 0.0001.

To further elucidate how microglia/macrophages and T cells mediate the outcome of RGCs, cytokine detection showed that IL-6 was increased in the ONT retina as well as in the injury-activated lymphocyte ONT retina co-culture, while depletion of microglia/macrophages considerably reduced IL-6 (**Figure 5A**). These results suggest that the increased IL-6 was originally secreted from microglia/macrophages in the ONT retina. However, TNF-α had different expression patterns (**Figure 5B**). TNF-α levels were higher in the co-culture of activated lymphocytes with the ONT retina, but not in either activated lymphocytes or ONT retina. TNF-α levels were initially reduced upon depletion of microglia/macrophages but recovered in the first 24 hours to reach levels similar to those seen with activated lymphocyte co-cultures. IL-4, IL-10, and IFN-γ were not detectable (data not shown).

Neutralization of IL-6, TNF-α, TNFR1, and TNFR2 all impaired the survival of RGCs compared with activated lymphocyte co-cultures (*P ≤* 0.0001). Following the addition of recombinant IL-6 or TNF-α (at final concentrations of 10, 50, 150, 200 ng/ml) to the cultured ONT retina, a dose-dependent enhancement of RGC viability was observed. IL-6 conferred enhanced survival from 100 ng/ml (*P*=0.012) to 150 ng/ml (*P*<0.0001), while 200 ng/ml dropped to no significant difference compared to 10 ng/ml. TNF-α showed a similar trend and reached a significant difference at 200 ng/ml (**Figures 5C, D**).

## Discussion

It has been long debated whether neuroinflammation benefits or impairs the survival of injured neurons. Results presented here indicate that both proinflammatory microglia/macrophage and peripheral T lymphocytes are activated and are beneficial for the restoration of injured neurons at the subacute stage of optic nerve injury. We further show that two microglia/macrophage-derived pro-inflammatory cytokines IL-6 and TNF-α play major roles.

In sterile CNS inflammation, the activation of microglia/macrophage is the first cellular response to acute and chronic insult (18). In this study, we found the upregulation of ED-1^+^ microglia/macrophage on the retina in the injury-activated lymphocytes but not in control lymphocytes coculture, indicating the interplay of CD4^+^CD25^+^ T cells and microglia/macrophages activation and proliferation. This is supported by the report that CD4^+^CD25^+^ T cells can induce the activation of macrophages (19). We find injury-activated macrophages are another stimulator for CD4^+^CD25^+^ T cells. However, we did not find significant differences in the survival of RGCs when deplete microglia/macrophages by clodronate, and even with the suppression of CD4^+^CD25^+^ T cells suggesting that compensatory mechanisms are at play.

Injury activates peripheral lymphocytes and promotes the generation of CD4^+^CD25^+^ T cells. It is reported that myelin basic protein (MBP) is one of the major antigens in CNS injury and specifically activates CD4^+^CD25^+^FoxP3^+^ T cells that exert neuroprotective effects (20, 21). Tregs (CD4^+^CD25^+^FoxP3^+^) are generally known as adaptive immunosuppressors. However, the role of Tregs in the injury of the central nervous system is controversial (22, 23). We do not differentiate CD4^+^CD25^+^FoxP3^+^ from CD4^+^CD25^+^ T cells due to the limited cells. As we detect the elevation of CD4^+^CD25^+^FoxP3^+^ in injury-activated T cells coculture, so we assume our interested cell group CD4^+^CD25^+^ is mixed with FoxP3^+^ and others.

The pro-inflammatory cytokines TNF-α and IL-6 suggesting produced by M1 microglia/macrophages (24). In our system, we confirmed that both IL-6 and TNFα are neuroprotective during neuroinflammation. IL-6 can be secreted by different cells, such as neurons, microglia, glial cells, and endothelial cells (25). However, the role of IL-6 in the CNS is controversial (26), with some studies showing that IL-6 promotes the generation of neuroprotective microglia and neurogenesis after brain injury (27), some studies suggesting that IL-6 increases neurodegeneration and inhibits axonal growth (28), and other studies suggesting that the different effects may be dependent on age and sex (29). TNF-α is a pleiotropic cytokine, some studies show its pretreatment is neuroprotective. It protects transplanted Neural progenitor cells from apoptosis in hypoxic-ischemic (HI) brain injury (30). Some studies show it induces apoptosis (31). In general, our findings support the notion that the pro-inflammation phase may be necessary to clear debris and set the stage for remodeling (4). Nevertheless, the time window to switch pro-inflammatory to anti-inflammatory is vital for long-term recovery.

Taken together, in the inflammation phase early after CNS injury, macrophages secrete pro-inflammatory factors, such as IL-6 and TNF-α, when peripheral lymphocytes infiltrate into the injury site *in vitro*, with the sum of activities resulting in neuroprotection.

## Data Availability Statement

The original contributions presented in the study are included in the article/Supplementary Material. Further inquiries can be directed to the corresponding author.

## Ethics Statement

The animal study was reviewed and approved by Shantou University Medical College Animal Experimentation Ethics Committee.

## Author Contributions

YZ designed the project. YG, ZL, and YZ performed the research. NR contributed liposomal clodronate. JG analyzed the data and YG and YZ prepared the manuscript. All authors contributed to the article and approved the submitted version.

## Funding

This work was supported by grants from the National Natural Science Foundation of China (No. 30671102) and the Natural Science Foundation of Guangdong Province, China (2015A030313444).

## Conflict of Interest

The authors declare that the research was conducted in the absence of any commercial or financial relationships that could be construed as a potential conflict of interest.

## Publisher’s Note

All claims expressed in this article are solely those of the authors and do not necessarily represent those of their affiliated organizations, or those of the publisher, the editors and the reviewers. Any product that may be evaluated in this article, or claim that may be made by its manufacturer, is not guaranteed or endorsed by the publisher.
